# Using Electronic Health Records to Examine Disease Risk in Small Populations: Obesity Among American Indian Children, Wisconsin, 2007–2012

**DOI:** 10.5888/pcd13.150479

**Published:** 2016-02-25

**Authors:** Emily J. Tomayko, Bethany A. Weinert, Liz Godfrey, Alexandra K. Adams, Lawrence P. Hanrahan

**Affiliations:** Author Affiliations: Emily J. Tomayko, University of Wisconsin, College of Agricultural and Life Sciences, Department of Nutritional Sciences, Madison, Wisconsin; Bethany A. Weinert, University of Wisconsin, School of Medicine and Public Health, Department of Pediatrics and Department of Family Medicine and Community Health, Madison, Wisconsin; Liz Godfrey, UW Health, Madison, Wisconsin; Alexandra K. Adams, University of Wisconsin, School of Medicine and Public Health, Department of Family Medicine and Community Health, Madison, Wisconsin.

## Abstract

**Introduction:**

Tribe-based or reservation-based data consistently show disproportionately high obesity rates among American Indian children, but little is known about the approximately 75% of American Indian children living off-reservation. We examined obesity among American Indian children seeking care off-reservation by using a database of de-identified electronic health records linked to community-level census variables.

**Methods:**

Data from electronic health records from American Indian children and a reference sample of non-Hispanic white children collected from 2007 through 2012 were abstracted to determine obesity prevalence. Related community-level and individual-level risk factors (eg, economic hardship, demographics) were examined using logistic regression.

**Results:**

The obesity rate for American Indian children (n = 1,482) was double the rate among non-Hispanic white children (n = 81,042) (20.0% vs 10.6%, *P* < .001). American Indian children were less likely to have had a well-child visit (55.9% vs 67.1%, *P* < .001) during which body mass index (BMI) was measured, which may partially explain why BMI was more likely to be missing from American Indian records (18.3% vs 14.6%, *P* < .001). Logistic regression demonstrated significantly increased obesity risk among American Indian children (odds ratio, 1.8; 95% confidence interval, 1.6–2.1) independent of age, sex, economic hardship, insurance status, and geographic designation.

**Conclusion:**

An electronic health record data set demonstrated high obesity rates for nonreservation-based American Indian children, rates that had not been previously assessed. This low-cost method may be used for assessing health risk for other understudied populations and to plan and evaluate targeted interventions.

## MEDSCAPE CME

Medscape, LLC is pleased to provide online continuing medical education (CME) for this journal article, allowing clinicians the opportunity to earn CME credit.

This activity has been planned and implemented in accordance with the Essential Areas and policies of the Accreditation Council for Continuing Medical Education through the joint sponsorship of Medscape, LLC and *Preventing Chronic Disease*. Medscape, LLC is accredited by the ACCME to provide continuing medical education for physicians.

Medscape, LLC designates this Journal-based CME activity for a maximum of 1 ***AMA PRA Category 1 Credit(s)™***. Physicians should claim only the credit commensurate with the extent of their participation in the activity.

All other clinicians completing this activity will be issued a certificate of participation. To participate in this journal CME activity: (1) review the learning objectives and author disclosures; (2) study the education content; (3) take the post-test with a 75% minimum passing score and complete the evaluation at www.medscape.org/journal/pcd; (4) view/print certificate.

**Release date: February 25, 2016; Expiration date: February 25, 2017**

### Learning Objectives

Upon completion of this activity, participants will be able to:

1. Compare the completeness of health records among American Indian vs non-Hispanic white children
2. Compare patterns of clinical care among American Indian vs non-Hispanic white children
3. Evaluate differences in the rate of obesity between American Indian and non-Hispanic white children
4. Assess risk factors for pediatric obesity

**EDITOR**

Rosemarie Perrin, Editor, *Preventing Chronic Disease*. 

Disclosure: Rosemarie Perrin has disclosed no relevant financial relationships.

**CME AUTHOR**

Charles P. Vega, MD, Clinical Professor of Family Medicine, University of California, Irvine

Disclosure: Charles P. Vega, MD, has disclosed the following relevant financial relationships.

Served as an advisor or consultant for: Allergan, Inc.; McNeil Consumer Healthcare; Takeda Pharmaceuticals North America, Inc. Served as a speaker or a member of a speakers bureau for: Shire Pharmaceuticals

**AUTHORS**

Emily J. Tomayko, PhD, RD, University of Wisconsin, College of Agricultural & Life Sciences, Department of Nutritional Sciences, Madison, Wisconsin

Disclosure: Emily J. Tomayko, PhD, RD, has disclosed no relevant financial relationships.

Bethany A. Weinert, MD, University of Wisconsin, School of Medicine and Public Health, Department of Pediatrics, Madison, Wisconsin

Disclosure: Bethany A. Weinert, MD, has disclosed no relevant financial relationships.

Liz Godfrey, MS, University of Wisconsin Health, Madison, Wisconsin

Disclosure: Liz Godfrey, MS, has disclosed no relevant financial relationships.

Alexandra K. Adams, MD, PhD, University of Wisconsin, School of Medicine and Public Health, Department of Family Medicine, Madison, Wisconsin

Disclosure: Alexandra K. Adams, MD, PhD, has disclosed no relevant financial relationships

Lawrence P. Hanrahan, PhD, University of Wisconsin, School of Medicine and Public Health, Department of Family Medicine, Madison, Wisconsin

Disclosure: Lawrence P. Hanrahan, PhD, has disclosed no relevant financial relationships.

## Introduction

Small population subgroups are often excluded from large-scale studies or surveys, making it difficult to assess health risk in these groups. Electronic health record (EHR) data sets may be well-suited to address these data gaps; a 2013 report to the US Department of Health and Human Services highlighted the feasibility of this approach and called for examination of underserved populations using this methodology (1). One such group is American Indian children who live outside of reservations or other tribal lands. Although data are available for American Indian children living on reservation through the Indian Health Service (IHS), less is known about children who live and seek health care outside of IHS. Data characterizing the differences in aspects of health and health care in this group and other subpopulations can lead to improvements in health care quality, evidence-based research, and public health approaches.

IHS is responsible for the health care of American Indian members of federally recognized tribes and serves approximately 2.2 million patients (2). However, approximately 2.9 million people self-identify as either American Indian or Alaska Native alone, and an additional 2.3 million self-identify as American Indian or Alaska Native in combination with other races, suggesting a significant proportion of this population may not receive medical care through IHS. Moreover, 78% of the American Indian and Alaska Native alone-or-in-combination population live outside of American Indian/Alaska Native reservations (3). Although nonreservation urban IHS clinics exist, most care that American Indians living outside of reservations receive is from the private sector (4).

Although little has been reported about American Indian children seeking care outside of reservations, the obesity prevalence among American Indian children living on reservations is known to be among the highest of all racial/ethnic groups (5–7) and may be related to socioeconomic and environmental factors. For example, the National Longitudinal Study of Adolescent to Adult Health (Add Health) included a subsample of American Indian teenagers that showed high rates of obesity associated with poverty and stress (8). However, the obesity prevalence or specific determinants of obesity in children seeking health care outside of reservation-based or IHS services are less well characterized, particularly among younger children.

The purpose of this study was to use the Public Health Information Exchange (PHINEX), a data set that links EHR data to community-level variables, to describe childhood obesity among American Indian children seeking health care in Wisconsin. The primary aim was to address a gap regarding the prevalence and risk profile of American Indian children seeking care outside of reservation areas. The secondary aim was to examine the usefulness of EHRs to examine disease risk in a small population as an alternative to resource-intensive surveys and data collection methods. We hypothesized this EHR-based approach would identify high rates of obesity among nonreservation-based American Indian children and demonstrate the usefulness of EHR data as a cost-effective and efficient resource for risk estimates among small populations.

## Methods

The University of Wisconsin (UW) PHINEX database links de-identified EHR data to community-level variables (eg, neighborhood socioeconomic and demographic variables), as described previously (9). PHINEX includes records of patient encounters that occurred from 2007 through 2012 at UW Health primary care clinics serving mostly south-central Wisconsin. Block group level data (ie, community-level data, typically containing 600–3,000 people) were extracted from 5-year estimates of the US Census American Community Survey 2007–2012 and from Esri Business Analyst (Esri). The study was reviewed and approved by the UW–Madison School of Medicine and Public Health institutional review board under Protocol M2009–1273, titled “Family Medicine/Public Health Data Exchange.”

Patient records were selected from the PHINEX database for patients who had at least one encounter from January 1, 2007, through December 31, 2012, while they were between the ages of 2 and 17 years and who were identified as American Indian. We included all patients who identified as American Indian for race and who indicated either Hispanic or non-Hispanic ethnicity. Patients in the same age range who were identified as non-Hispanic white were included as the reference group. In the PHINEX database, 98.7% of patients selected only one response for race (with ethnicity reported as a separate category). Race information was listed as “unknown” for 5.2% of patients, and missing race information was less than 1%. Although UW Health clinics may differ in the way race/ethnicity data are collected (eg, collected at registration desk vs in-office by nurse), the standard practice is for the patient (or parent or guardian) to self-identify race/ethnicity on the clinic intake form. We excluded patient records that did not meet age or race/ethnicity criteria and performed complete case analysis for body mass index (BMI) (kg/m^2^) and other individual-level variables.

Patient data were collected by clinic staff during documented primary care encounters (family medicine and pediatrics). Variables included in this study were age, BMI, sex, self-reported or parent-reported race/ethnicity, number and type of encounters, and insurance type. Number of encounters was calculated as visits per patient year (number of visits divided by number of years for which patients had at least one visit during the study period). BMI measures for children were plotted on age-specific and sex-specific growth charts to determine BMI percentile according to the CDC 2000 charts (<5th percentile = underweight, 5th to <85th percentile = normal weight, 85th to <95th percentile = overweight, ≥95th percentile = obese) (10). If multiple patient encounters were available, the most recently recorded BMI was used.

Before de-identification, patients were linked to census block groups by using their geocoded address of residence. Two community-level variables were used: geographic designation (ie, urban, rural, suburban) and economic hardship index (EHI) score by census block group as a measure of community-level socioeconomic status (11). The geographic designation was based on the acquisition of census block groups using geocoded patient addresses. Esri’s Tapestry demographic segmentation methodology was then applied, which divides US residential areas into distinctive segments on the basis of socioeconomic and demographic characteristics to provide an accurate description of neighborhoods. The calculation of EHI has been described previously in Appendix 1 of Nathan and Adams (12) and was normalized for all Wisconsin block groups for this analysis. The economic hardship index was calculated at the census block group level and consists of 6 measures: crowded housing (percentage of housing units with more than 1 person per room), poverty (percentage of households below the federal poverty level), unemployment (percentage of people aged 16 years or older who are unemployed), education (percentage of people aged 25 years or older without a high school education), dependency (percentage of population younger than 18 years or older than 64 years), and per capita income. Scores can range from 0 to 100, with 100 indicating the highest hardship (ie, crowded housing, poverty, unemployment, large number of dependents, low per capita income, and low education level) (12).

Statistical analysis was performed using SAS software, version 9.3 (SAS Institute, Inc). Variables were compared between the American Indian population and the non-Hispanic white population (reference population) within the PHINEX database using Pearson χ^2^ statistical testing or the Wilcoxon test to examine group differences. Odds ratios (ORs) were calculated using stepwise logistic regression adjusted for EHI, insurance status, ethnicity, sex, age, and geographic designation. A drop-in-deviance test was used to test the effect of race: the full model included an effect for American Indian as well as interaction terms between American Indian and covariates, and the reduced model included only covariates. The Akaike information criterion was used for model selection.

## Results

For all available records, no data were missing for age, sex, or insurance status. BMI measurement in the electronic record was more likely to be missing for American Indian patients than for non-Hispanic white patients (18.3% vs 14.6%, *P* < .001). There were no differences between American Indian and non-Hispanic white patients in missing data related to EHI (9.3% vs 8.6%, *P* = .32) or geographic designation (9.3% vs 8.5%, *P* = .32).

The study included 1,482 American Indian patients and 81,042 non-Hispanic white patients with documented primary care visits between 2007 and 2012. Of the American Indian patients, 66% were Hispanic. American Indian children were 1.4% of the total population within the PHINEX database. All demographic variables differed significantly (*P* < .001) between American Indian and non-Hispanic white populations except for sex (Table 1). A higher percentage of children aged 5 to 11 years were American Indian (51.4% vs 41.9%). More non-Hispanic white children had commercial insurance (83.7%) than American Indian children (53.7%). More American Indian children than non-Hispanic white children had Medicaid (42.1% vs 14.7%) or no health insurance (4.1% vs 1.6%). More American Indian children had an EHI classification greater than 25 (52.8% vs 40.6%) than non-Hispanic white children, indicating a higher degree of economic hardship.

**Table 1:** Demographic Characteristics, American Indian and Non-Hispanic White Children,^a^ University of Wisconsin Clinics, 2007–2012

Characteristic	American Indian, n (%)	Non-Hispanic White, n (%)
**Total**	1,482 (1.4)	81,042 (79.3)^b^
**Age, y**		
2–4	266 (18.0)	16,784 (20.7)
5–11	762 (51.4)	33,945 (41.9)
12–17	454 (30.6)	30,313 (37.4)
**Male**	776 (52.4)	42,325 (52.2)
**Body mass index^c^**		
Underweight	28 (1.9)	2,399 (3.0)
Normal weight	668 (45.1)	47,930 (59.1)
Overweight	219 (14.8)	10,289 (12.7)
Obese	296 (20.0)	8,576 (10.6)
Data missing	271 (18.3)	11,848 (14.6)
**Health insurance**		
Commercial	796 (53.7)	67,839 (83.7)
Medicaid	624 (42.1)	11,879 (14.7)
Medicare	1 (0.1)	12 (0.01)
No insurance	61 (4.1)	1,307 (1.6)
**Economic hardship index^d^**		
<20	143 (9.6)	8,190 (10.1)
20–<25	419 (28.3)	33,063 (40.8)
≥25	782 (52.8)	32,862 (40.6)
Data missing	138 (9.3)	6,927 (8.6)
**Geographic designation**		
Urban	655 (44.2)	15,211 (18.8)
Suburban	538 (36.3)	43,267 (53.4)
Rural	151 (10.2)	15,641 (19.3)
Data missing	138 (9.3)	6,923 (8.5)

a Identified by using the Public Health Information Exchange (PHINEX), a database that links electronic health records collected at University of Wisconsin clinics with community-level census variables.

b African-American, Asian, and other groups account for the remaining 19.3% of the PHINEX database.

c Body mass index (kg/m^2^) was calculated from height and weight measured on the same day. All BMI values were plotted on age-specific and sex-specific growth charts to determine BMI percentile according to the CDC 2000 charts as follows: <5th percentile = underweight, 5th to <85th percentile = normal weight, ≥85th to <95th percentile = overweight, and ≥95th percentile = obese (10). If multiple patient encounters were available, the most recently recorded BMI was used.

d The economic hardship index is calculated at the census block group level and consists of 6 measures: crowded housing (percentage of housing units with fewer than 1 person per room), poverty (percentage of households below the federal poverty level), unemployment (percentage of people aged 16 years or older who are unemployed), education (percentage of people aged 25 years or older without a high school education), dependency (percentage of population younger than 18 years or older than 64 years), and per capita income. Scores can range from 0 to 100, with 100 indicating the highest hardship (12).

American Indian children had slightly fewer total clinic visits per person-year than non-Hispanic white children (mean [SD], 2.5 [1.9] vs mean [SD], 2.6 [1.9]; *P* < .01) (Table 2). Moreover, despite the similar number of visits per person-year, American Indian children had significantly fewer well-child visits (55.9% vs 67.1%, *P* < .001). The clinic visits for American Indian and non-Hispanic white children appeared to be of similar medical complexity; American Indian children had a comparable number of diagnoses per visit compared with non-Hispanic white children (mean [SD], 1.9 [0.7] for both; *P* = .20).

**Table 2:** Characteristics of Clinic Visits, American Indian and Non-Hispanic White Children, Taken From Electronic Health Records, University of Wisconsin Health Clinics, 2007–2012^a^

Clinic Visit	American Indian	Non-Hispanic White	*P* Value
Visits per child per year, mean (SD)	2.5 (1.9)	2.6 (1.9)	<.01
Diagnoses per visit, mean (SD)	1.9 (0.7)	1.9 (0.7)	.20
Well-child visits, n (%)	767 (55.9)	55,043 (67.1)	<.001

a Pearson χ^2^ or the Wilcoxon test was used to compare American Indian and non-Hispanic white children.

American Indian children had nearly double the rate of obesity compared with the non-Hispanic white population (20.0% vs 10.6%, *P* < .001) and a higher rate of overweight (14.8% vs 12.7%, *P* < .001) (Table 1). A drop-in-deviance test indicated a significant effect of American Indian race on obesity (*P* < .001). The Akaike information criterion indicated that a model with only a main effect for American Indian race provided a better fit than a model with American Indian race as well as interaction terms between American Indian race and the covariates. In a model that included race/ethnicity, age, sex, economic hardship, insurance status, and geographic designation, logistic regression analysis for obesity showed the odds of obesity were significantly higher for children self-identified as American Indian, those for whom Medicaid or Medicare was listed for health insurance, children living in a census block group with an EHI of 25 or greater, children aged 12 to 17, and male children (Table 3).

**Table 3:** Obesity Risk in a Multivariate Model of American Indian and Non-Hispanic White Children, University of Wisconsin Health Clinics, 2007–2012

Variable	Adjusted Odds Ratio (95% CI)	*P *Value
**Economic hardship index^a^**		
<20	0.48 (0.44–0.53)	<.001
20–<25	0.67 (0.64–0.71)	<.001
≥25	1 [Reference]	
**Health insurance **		
Commercial	1 [Reference]	
Medicare or Medicaid	1.74 (1.63–1.84)	<.001
No insurance	1.11 (0.85–1.45)	.43
**Race/ethnicity**		
American Indian	1.85 (1.60–2.14)	<.001
Non-Hispanic white	1 [Reference]	
**Sex**		
Female	1 [Reference]	
Male	1.31 (1.25–1.38)	<.001
**Age, y**		
2–4 years	0.49 (0.46–0.53)	<.001
5–11 years	0.83 (0.78–0.87)	<.001
12–17	1 [Reference]	
**Geographic designation**		
Urban	1 [Reference]	
Suburban	0.86 (0.81–0.92)	<.001
Rural	0.93 (0.86–0.996)	.04

Abbreviation: CI, confidence interval.

a The economic hardship index is calculated at the census block group level and consists of 6 measures: crowded housing (percentage of housing units with fewer than 1 person per room), poverty (percentage of households below the federal poverty level), unemployment (percentage of people aged 16 years or older who are unemployed), education (percentage of people aged 25 years or older without a high school education), dependency (percentage of population younger than 18 years or older than 64 years), and per capita income. Scores can range from 0 to 100, with 100 indicating the highest hardship (12).

## Discussion

Our study demonstrates the usefulness of an electronic health record database to analyze disease risk in a small population. By using this method, we addressed a gap in the literature by examining childhood obesity in American Indian children receiving care outside of IHS or tribal clinics, a population typically excluded from national and local health surveys.

By using the PHINEX database, our analysis demonstrated a higher rate of overweight and obesity in American Indian children compared with a reference sample of non-Hispanic white children seen at the same clinics. The obesity disparity was markedly greater than the disparity in overweight between non-Hispanic white and American Indian children. These patterns are similar to those identified in a data set of reservation-based American Indian children in Wisconsin (aged 3–8 y) of 20.0% overweight and 25.2% obese (13). In addition, a comprehensive review of American Indian children in tribal communities nationwide identified high rates of both overweight and obesity, although the rates were highly variable among populations (14). As in other minority communities, the reasons for the increased prevalence among American Indians are complex and include poverty, racism, historic trauma, rural isolation, urban loss of community, stress, lack of access to healthy foods and physical activity opportunities, and safety issues that may prevent physical activity (2,15–18). In addition, American Indian children are at increased risk for obesity-related chronic diseases such as diabetes and cardiovascular diseases, which are observed at high rates in this population (19,20).

In our study, American Indian children were also more likely to have Medicaid, to live in urban settings, and to have higher economic hardship than non-Hispanic white children. In our regression analysis, self-report as American Indian, EHI classification of 25 or greater, and use of Medicaid or Medicare insurance significantly increased the odds of obesity. Other previously reported risk factors also were found to be significant in our analysis, including increased odds of obesity for older (12–17 y) and male children (21).

Our previous work has demonstrated the PHINEX database to be both representative of the state of Wisconsin (9) and able to determine obesity rates with precision, particularly in comparison to large national data sets, such as the National Health and Nutrition Examination Survey (NHANES) (22). Although comparison rates are available for non-Hispanic white children in NHANES, American Indian children are typically included only in the “All Race” category in NHANES reports from 1999 through 2012 (23,24). Data from the 2011 Pediatric Nutrition Surveillance Survey (PedNSS) reported national rates of overweight of 20.1% and obesity rates of 20.8% for American Indian/Alaska Native children older than 2 years of age (6). However, these data were collected primarily from the Special Supplemental Nutrition Program for Women, Infants, and Children (WIC) records (87.0% of records), which included only low-income children aged 5 years or younger and therefore excluded older children and children from higher-income families. Another difficulty with using the PedNSS as a reference population is that American Indian data were provided by 7 reservation-based tribal organizations nationwide, and nonreservation-based American Indian children were not sampled.

IHS collects data on childhood overweight and obesity only for patients who use IHS clinics (ie, reservation or near-reservation–based care), whereas many other studies that included American Indian data were conducted in small geographic areas or represent nonsurveillance studies (14). Add Health focused on teenagers and found that 76.8% of participants in their subsample were overweight or obese. The Add Health study required blood sample collection and direct measurement of BMI, which can be costly and time-consuming outside of a health care setting (8). Therefore, EHR-based approaches like the one described in our study represent an effective approach to monitor childhood obesity rates in nonreservation-based American Indian children and other small populations.

A previous report identified few data sets for American Indian populations with sample sizes over 200 and cited, among multiple barriers, geographic distribution of people living in nonreservation-based urban or rural areas and failure to collect race/ethnicity information for several large data sets (25). Rather than increasing the sample size in large federal studies or other resource-intensive approaches, using EHRs to analyze a small population has several advantages. In addition to being a rapid and inexpensive method of data collection, EHRs also provide the capacity for data set linkage and the ability to access typically excluded populations, such as the nonreservation-based American Indian children described here. For example, future research could include a de-identified database linking child and maternal health records within the PHINEX database, because maternal BMI is known to be a predictor of a child’s weight. In addition, this database could be used for other groups, such as Asian minority populations and refugee groups. EHRs also offer opportunities for quality improvement in health care, such as identifying the need for consistent obesity screening protocols. Moreover, EHR data sets such as PHINEX provide a larger sample than could be easily collected otherwise. EHR data sets ultimately allow for greater surveillance of population health and risk factors at the local level (eg, census block group), which provides an unprecedented opportunity for communities to detect local health hot spots and address them with local community-level interventions. IHS data typically are anonymous to protect tribal identity, thereby preventing the examination of this local variation. Comparison of the children included in this study who sought care outside of IHS with children seeking care at IHS or tribal clinics would allow for a more complete examination of obesity determinants and represents a collaboration we hope to pursue in the future.

Our study has several limitations. The overall sample size of American Indian children was considerably smaller than that of non-Hispanic white children; however, the events of interest, overweight and obesity, were not rare events, and it is therefore unlikely that our rates were biased (26). Our analyses relied on self-reported racial and ethnic classification, and protocols for collecting this information vary among the clinics; previous reports have suggested that misclassification of American Indian patients as another race or ethnicity is a significant problem (27–29). Despite these facts, our analysis included approximately 1,500 American Indian children and is the largest analysis examining childhood obesity in this particular population to date. Patients with missing data were dropped from the analysis, and this could potentially bias results. In addition, BMI was more likely to be missing for American Indian children than for non-Hispanic white children in the PHINEX database, which may be because American Indian children had significantly fewer well-child visits during which BMI is usually calculated. This information could be used to educate clinicians to improve the collection and reporting of these data and to reinforce the importance of supporting American Indian families in seeking preventive health care (eg, well-child visits). Although several factors probably contribute to the significantly higher rates of obesity for American Indian children than for non-Hispanic white children, including socioeconomic status and environmental factors, no causative factors could be determined from this study. Finally, there may have been selection bias toward children who are more likely to attend clinic visits, such as children who have family members with sufficient schedule flexibility and reliable transportation. Despite these limitations, increased obesity prevalence was found in this American Indian population and at a similar magnitude found in previous studies.

More research is needed to address important concepts of race and place in determining risk for childhood obesity and other health outcomes. It remains unclear whether American Indian children seeking care outside of tribal or IHS clinics more closely resemble children in the geographic area in which they live (such as through shared experience of economic hardship measured at the community or census block level) or align more closely with their reservation-based counterparts. This study also emphasizes the importance of standardizing the collection of race and ethnicity information in electronic medical records to decrease misclassification and missing information for future analysis.

EHR-based analysis can inform clinicians and other health care providers regarding disparities, not only in health outcomes but also in the provision of health care (eg, attendance at well-child visits) for underserved populations while directing future intervention research to address these issues. By using EHRs, we demonstrated that American Indian children seeking care outside of IHS or tribal clinics in Wisconsin experience overweight and obesity at significantly higher rates than non-Hispanic white children. Use of EHRs is a cost-effective way to examine health risks in other understudied populations.

## Figures and Tables

**Table:** 

**1. The activity supported the learning objectives.**				
**Strongly Disagree**				**Strongly Agree**
1	2	3	4	5
**2. The material was organized clearly for learning to occur.**				
**Strongly Disagree**				**Strongly Agree**
1	2	3	4	5
**3. The content learned from this activity will impact my practice.**				
**Strongly Disagree**				**Strongly Agree**
1	2	3	4	5
**4. The activity was presented objectively and free of commercial bias.**				
**Strongly Disagree**				**Strongly Agree**
1	2	3	4	5

## Post-Test Information

To obtain credit, you should first read the journal article. After reading the article, you should be able to answer the following, related, multiple-choice questions. To complete the questions (with a minimum 75% passing score) and earn continuing medical education (CME) credit, please go to http://www.medscape.org/journal/pcd. Credit cannot be obtained for tests completed on paper, although you may use the worksheet below to keep a record of your answers. You must be a registered user on Medscape.org. If you are not registered on Medscape.org, please click on the "Register" link on the right hand side of the website to register. Only one answer is correct for each question. Once you successfully answer all post-test questions you will be able to view and/or print your certificate. For questions regarding the content of this activity, contact the accredited provider, CME@medscape.net. For technical assistance, contact CME@webmd.net. American Medical Association's Physician's Recognition Award (AMA PRA) credits are accepted in the US as evidence of participation in CME activities. For further information on this award, please refer to http://www.ama-assn.org/ama/pub/about-ama/awards/ama-physicians-recognition-award.page. The AMA has determined that physicians not licensed in the US who participate in this CME activity are eligible for ***AMA PRA Category 1 Credits*** ™. Through agreements that the AMA has made with agencies in some countries, AMA PRA credit may be acceptable as evidence of participation in CME activities. If you are not licensed in the US, please complete the questions online, print the AMA PRA CME credit certificate and present it to your national medical association for review.

## Post-Test Questions

### Study Title: Innovative Use of Electronic Health Records to Examine Disease Risk in Small Populations

**CME Questions**

1. You are opening a practice in a diverse community with a large American Indian population, and you want to identify community health issues which might affect American Indian children in particular. In the current study by Tomayko and colleagues, which of the following data points was more likely to be missing from the record of American Indian vs non-Hispanic white children?
  - Geographic designation
  - Body weight
  - Body mass index
  - Socioeconomic data
2. What was the *most* significant pattern in the type of care provided to American Indian vs non-Hispanic white children in the current study?
  - American Indian children had more total clinic visits per year compared with non-Hispanic white children
  - American Indian children had fewer well-child visits per year compared with non-Hispanic white children
  - American Indian children had higher complexity outpatient visits compared with non-Hispanic white children
  - American Indian children were more likely to be hospitalized compared with non-Hispanic white children
3. What was the approximate increase in the rate of obesity in comparing American Indian vs non-Hispanic white children in the current study?
  - <3%
  - 32%
  - 100%
  - 450%
4. Which of the following variables was a significant risk factor for obesity in the current study?
  - Self-identification as American Indian
  - Female gender
  - Age younger than 7 years
  - Lack of health insurance

**Evaluation**
